# The role of PTEN as a cancer biomarker

**DOI:** 10.18632/oncoscience.296

**Published:** 2016-03-03

**Authors:** Nuala McCabe, Richard D. Kennedy, Kevin M. Prise

**Affiliations:** Center for Cancer Research & Cell Biology, Queens University Belfast, UK

**Keywords:** PTEN, predictive biomarker, tumour suppressor Gene

## THE PTEN TUMOUR SUPPRESSOR GENE

The phosphatase and tensin homologue, PTEN, was identified in 1997 and later found to be frequently disrupted in multiple sporadic tumour types and targeted by germline mutations in patients with cancer predisposition syndromes such as Cowden disease [1]. The principal catalytic function of PTEN is to dephosphorylate phosphatidylinositol-3,4,5-trisphosphate (PtdIns(3,4,5) P3), which is a potent activator of 3-phosphoinositidedependent kinase (PDK) and AKT. As a consequence, loss of PTEN function leads to increased levels of PtdIns(3,4,5)P3 and activation of the phosphoinositide 3-kinase (PI3K)–AKT pathway which stimulates cell growth and survival. Additionally, recent data demonstrate that nuclear PTEN has now been demonstrated to maintain genomic stability through regulation of RAD51, a key protein involved in double-strand break (DSB) repair and stabilisation of replication fork during replication stress [2]. These distinct functions of PTEN and associated cancer predisposing mutations, has caused great interest in PTEN as a cancer biomarker.

## PTEN AS A BIOMARKER IN ESTIMATING RISK

Germline mutations of PTEN are the underlying genetic causes of related disorders clinically referred to as PTEN hamartoma syndromes (PHTS) including Cowden syndrome. Mutations responsible for these syndromes result in a non-functional or absent protein, which causes uncontrolled cell growth, leading to tumour (either benign or malignant) growth. Additionally these patients have a predisposition for cancer with increased lifetime risks for breast (85%), thyroid (35%), renal (33%), and endometrial (28%) cancers, colorectal cancers (9%) and melanoma (6%) [3].

## PTEN AS A PROGNOSTIC BIOMARKER

The cloning of the PTEN gene to human chromosome 10q23.3, was accompanied by detection of various types of mutations including homozygous deletion, frameshift, inframe deletion, truncation and point mutation [1]. Additionally post-translational modifications including phosphorylation, acetylation, methylation, oxidation have also been implicated in the loss of PTEN function and in the initiation of tumourigenesis [4]. Whether through mutation or epigenetic regulation, the loss or aberration of the PTEN gene/protein can have prognostic impact in the cancers which manifest these alterations. *PTEN* loss has been shown to be associated with poor outcome in a variety of cancers including prostate cancer (PCa), glioblastoma and colorectal cancer [1, 4]. For example approximately 2–14% of prostate cancer specimens were shown to harbour PTEN mutations, and 12–41% have copy number loss [4]. It has been demonstrated that there was a higher frequency of *PTEN* loss in more advanced castrate resistant PCa (CRPC) cases and that *PTEN* loss was associated with shorter progression-free survival time [4].

## PTEN AS A PREDICTIVE BIOMARKER

PTEN has been associated with response to conventional standard of care chemotherapy. PTENnegative tumours have also been shown to have shorter survival in the post-docetaxel abiraterone treatment setting compared with cases with preserved *PTEN* expression [5]. Additionally PTEN loss has previously been reported to be prognostic for outcome following radiotherapy in prostate cancer [4]. PTEN expression also shows promise as a predictive marker for targeted therapeutic agents including anti-EGFR mAbs [6], trastuzumab-based chemotherapy in breast cancer [7]. Additionally PTEN loss has been demonstrated to induce sensitivity to PARP1/2 inhibition in cell line models, however recent findings from TOPARP trial (NCT01682772) indicate that PTEN loss does not confer sensitivity to PARP inhibition using olaparib [8] suggesting that a greater understanding of the role of PTEN in DNA repair and therefore PARP inhibitor sensitivity will need to be gained. Additionally our lab has demonstrated a function for PTEN in controlling oxidative DNA damage was recently demonstrated and therapeutically exploited using an ataxia telangiectasia mutated (ATM) inhibitor [9]. We have demonstrated that the sensitivity of PTEN null cells to ATM inhibition was dependent of the generation of oxidative DNA damage, and independent of RAD51 function, suggesting further nuclear roles for PTEN.

The utility of using PTEN as a biomarker of prognosis or predictor for drug response clearly needs further investigation. Only through a greater understanding of the function played by PTEN in regulating various biological functions will its role as a biomarker be fully realised. Additionally it will be imperative to evaluate the monopoly of cancer associated mutations and posttranslational modifications which target these functions in clinical samples. This will be important in defining the best methods for detecting PTEN aberration for best clinical impact.
